# A “One Health” Approach to Address Emerging Zoonoses: The HALI Project in Tanzania

**DOI:** 10.1371/journal.pmed.1000190

**Published:** 2009-12-15

**Authors:** Jonna A. K. Mazet, Deana L. Clifford, Peter B. Coppolillo, Anil B. Deolalikar, Jon D. Erickson, Rudovick R. Kazwala

**Affiliations:** 1 Wildlife Health Center, School of Veterinary Medicine, University of California, Davis, California, United States of America; 2 Wildlife Conservation Society, Bozeman, Montana, United States of America; 3 Department of Economics, University of California, Riverside, California, United States of America; 4 Rubenstein School of Environment and Natural Resources, University of Vermont, Burlington, Vermont, United States of America; 5 Department of Veterinary Medicine and Public Health, Sokoine University of Agriculture, Morogoro, Tanzania; United States of America

## Abstract

Jonna Mazet and colleagues describe their work in the Tanzania-based HALI Project, which adopts the “One Health” approach to address emerging zoonoses and that recognizes the interconnectedness of human, animal, and environmental health.

## Need for Integrated Health Approaches

Every day thousands of children and adults die from underdiagnosed diseases that have arisen at the human–animal–environment interface, especially diarrheal and respiratory diseases in developing countries [1],[2]. Explosive human population growth and environmental changes have resulted in increased numbers of people living in close contact with wild and domestic animals. Unfortunately, this increased contact together with changes in land use, including livestock grazing and crop production, have altered the inherent ecological balance between pathogens and their human and animal hosts. In fact, zoonotic pathogens, such as influenza and SARS (severe acute respiratory syndrome), account for the majority of emerging infectious diseases in people [3], and more than three-quarters of emerging zoonoses are the result of wildlife-origin pathogens [4]. While zoonoses represent a significant emerging threat to public health, many of these diseases, such as diarrheal diseases arising from poor water sanitation, are neglected by funding agencies [5].

### Role of Water and Natural Resource Limitation

Nowhere in the world are these health impacts more important than in developing countries, where daily workloads are highly dependent on the availability of natural resources [6],[7]. Water resources are perhaps most crucial, as humans and animals depend on safe water for health and survival, and sources of clean water are dwindling due to demands from agriculture and global climate change. As water becomes more scarce, animals and people are squeezed into smaller and smaller workable areas. Contact among infected animals and people then increases, facilitating disease transmission. Water scarcity also means that people and animals use the same water sources for drinking and bathing, which results in serious contamination of drinking water and increased risk of zoonotic diseases. In addition, poor sanitation and animal management can result in fecal contamination of both animal and human food. When this situation is complicated by high HIV/AIDS prevalence, the impacts of otherwise minimally virulent or difficult-to-transmit pathogens can be catastrophic to families and entire communities, and ultimately to the environment through impacts on human capacity, natural resource management, and land use [8].

The conditions of land-use change, water scarcity, and overlapping human, livestock, and wildlife populations are particularly prevalent in rural Africa and near remaining wildlands. Human population in sub-Saharan Africa doubled between 1975 and 2001 [9], and the African Population and Health Research Center predicts another doubling from 2008 levels to 1.9 billion by 2050. Such rapid population growth and consequent demands for natural resources are making African wildlands increasingly vulnerable to conversion to other land uses, such as logging, agriculture, and pasturage. A recent analysis by Wittermyer et al. [10] found that average annual population growth rates were higher in buffers to protected areas than in rural areas in Africa and Latin America. Protected areas provide some of the last supplies of ecosystem goods and services for expanding human populations, including firewood, bush meat, clean water, medicinal plants, and areas of safety during civil strife. Their porous edges also provide refuge for the vectors of zoonotic disease transmission.

### The One Health Approach

**Figure 1:**
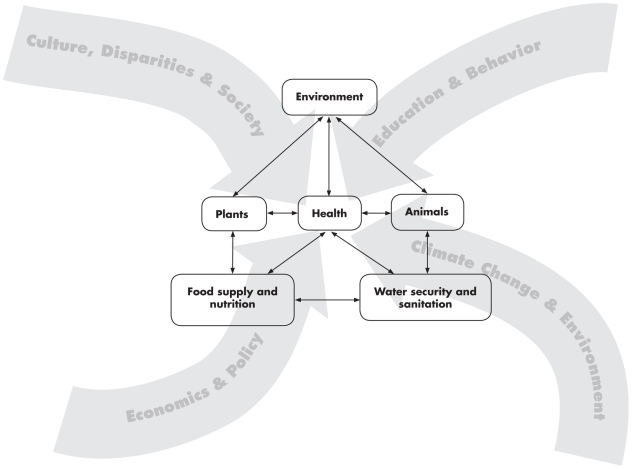
The local and global influences impacting human health, including the interdependence of people, animals, plants, and the environment, and the associated food and water availability, safety, and security. (Graphic artist credit: A. Kent).

The interconnectedness of human, animal, and environmental health is at the heart of One Health, an increasingly important prism through which governments, NGOs (nongovernmental organizations), and practitioners view human health [11]. An important implication of the One Health approach is that integrated policy interventions that simultaneously and holistically address multiple and interacting causes of poor human health—unsafe and scarce water, lack of sanitation, food insecurity, and close proximity between animals and humans—will yield significantly larger health benefits than policies that target each of these factors individually and in isolation. By its very nature, the One Health approach is transdisciplinary, since it is predicated on agricultural scientists, anthropologists, economists, educators, engineers, entomologists, epidemiologists, hydrologists, microbiologists, nutritionists, physicians, public health professionals, sociologists, and veterinarians working collaboratively to improve and promote both human and animal health. Figure 1 depicts the relationship among health, safe water, and food supply and their dependence upon plants, animals, and the environment, as well as the influences that interact to affect human health. This complexity necessitates a collaborative approach among professionals from multiple disciplines for the design of effective interventions.

## Applying the One Health Paradigm

### The HALI Project

Assessing and reducing the impacts of zoonotic diseases and resource limitation on health and livelihoods requires governments, NGOs, and academic institutions to work with citizens to develop interventions that are cost effective, sustainable, and conservation minded. In 2006, the Health for Animals and Livelihood Improvement (HALI; http://haliproject.wordpress.com/) project was initiated to test the feasibility of the One Health approach in rural Tanzania and to find creative solutions to these problems by investigating the impact of zoonotic disease on the health and livelihoods of rural Tanzanians living in the water-limited Ruaha ecosystem. HALI, from the Swahili word for state of health, addresses these complex disease and natural resources issues on a platform that recognizes that the health of domestic animals, wildlife, and people is inextricably linked to the ecosystem and natural resources on which all depend [12].

The Ruaha landscape is one of Tanzania's largest wild areas, covering a region larger than Denmark (>45,000 km^2^). This sprawling ecosystem is of extraordinary conservation significance and supports approximately 30,000 elephants (*Loxodonta africana*) and the continent's third largest population of critically endangered African wild dogs (*Lycaon pictus*) [13]. The socioeconomic importance of the Ruaha region rivals its biological significance, as virtually all communities depend entirely on the natural resource base, and agriculture accounts for about 80% of these livelihoods [14]. This importance is immediately apparent at the village level, where livestock are widespread, abundant, and central to traditional natural resource management. Unfortunately, livestock-dependent households are among the poorest in the nation [15]. This local poverty fuels the demand for illegal wildlife hunting for meat, another known driver for disease emergence [16].

Zoonotic diseases known to be of public health importance, such as rabies and Rift Valley fever, are present in wildlife, domestic animals, and people in Tanzania [17]; however, the role of underdiagnosed diseases, such as bovine tuberculosis (BTB), has only just begun to be characterized [18],[19]. Nearly 40,000 new cases of tuberculosis (human, bovine, or atypical strain) are diagnosed per year in Tanzania [20], with anywhere from 21% to 77% of Tanzanian tuberculosis patients also infected with HIV [21]. The extrapulmonary form of tuberculosis (EPTB) in people, often associated with BTB infection from animals, accounts for 20% of the reported cases in Tanzania [20]. Therefore, bovine tuberculosis became a focal disease for the HALI project due to its high livestock prevalence [22], wildlife data paucity, and the large, susceptible HIV-infected human population living in close association with livestock and wildlife. Additional priorities for HALI were determined through gender-balanced interviews with affected communities, including village chair people; leaders of agricultural, water, and women's cooperatives; and heads and members of pastoralist households. An overwhelming consensus emerged from follow-up stakeholder meetings of diverse communities, including multiple levels of government (including public hospital physicians), nonprofit organizations, academic institutions, and citizens:

*A significant proportion of the rural population in the Ruaha landscape is affected by diseases impacted by water supply, and these diseases are affecting health, agricultural productivity, food security, and biodiversity in the region.*

### HALI's Multilevel Approach

**Figure 2:**
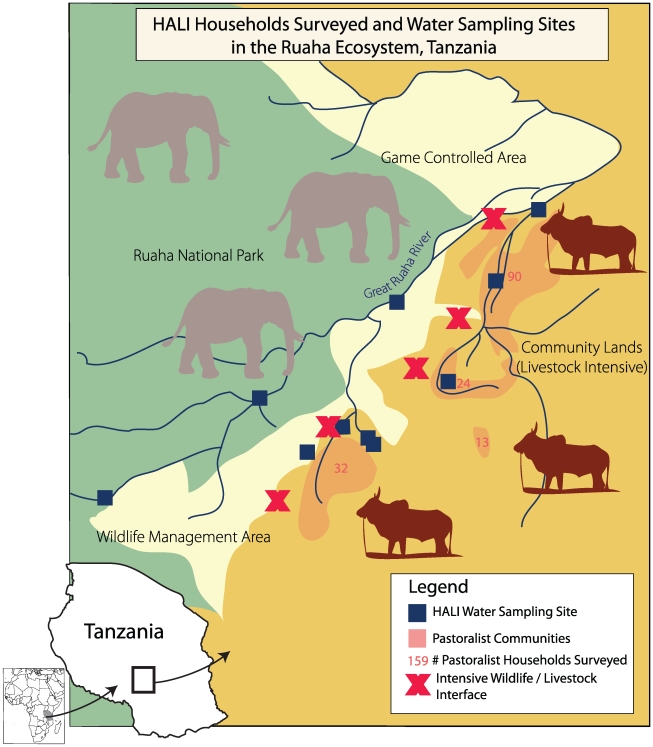
Map of the HALI Project study site in the Ruaha ecosystem, Tanzania. (Graphic artist credit: A. Kent).

Accordingly, the HALI project is assessing the impact of the interactions between water and disease in the Ruaha ecosystem by simultaneously investigating the medical, ecological, socioeconomic, and policy issues driving the system (Table 1). The map in Figure 2 illustrates our multilevel approach, which includes: testing of wildlife, livestock, and their water sources for zoonotic pathogens and disease; environmental monitoring of water quality, availability, and use; assessing wildlife population health and demography; evaluating livestock and human disease impacts on livelihoods of pastoralist households; examining land and water use impacts on daily workloads and village economies; introducing new diagnostic techniques for disease detection; training Tanzanians of all education levels about zoonotic diseases; and developing new health and environmental policy interventions to mitigate the impacts of zoonotic diseases. Perhaps most importantly, the HALI project is examining these issues in a common framework with specific emphasis on the interactions between them, instead of attempting to isolate a single issue.

**Table 1:** The HALI Project's multilevel approach to assessing the impact of the interactions between water and disease in the Ruaha ecosystem by simultaneously investigating the medical, ecological, socioeconomic, and policy issues driving the system.

System Drivers	Objective	Activities
***Medical***	Assess wildlife, livestock, and their water sources for zoonotic pathogens and disease including bovine tuberculosis, brucellosis, *Salmonella*, *Cryptosporidium*, *Giardia*, *E. coli*, and *Campylobacter*.	• 70 wildlife samples tested
		• 1,368 live cattle from 102 pastoralist households tested
		• 228 livestock carcasses tested
		• Ten water sources sampled monthly for 2 years
	Evaluate pastoralists' perceptions about disease impacts and risk of transmission from animals and water.	• 159 household surveys estimating disease impacts and examining transmission risk factors (subset resampled seasonally)
	Introduce new diagnostic techniques for disease detection.	• Transfer of five technologies between University of California, Davis (US) and Sokoine University of Agriculture (Tanzania)
	Train Tanzanians of ALL education levels about zoonotic disease.	• Community outreach to over 950 local people
		• Training for 24 game scouts and technicians
		• Four honors bachelor and extern projects
		• Two masters theses
***Ecological***	Environmental monitoring of water quality and availability	• Ten water sources sampled monthly for 2 years
	Assess wildlife population health and demography	• Surveys in association with Wildlife Conservation Society, Tanzania National Parks, and the local community managing wildlife
	Examine landscape-level risk factors for disease	• Integration of spatial data on wildlife and livestock density, regions of water scarcity, and land use regimes
***Socioeconomic***	Evaluate livestock and human disease impacts on livelihoods of pastoralist households	• 159 household surveys examining economic risk factors (subset resampled seasonally)
	Examine land and water use impacts on daily workloads and village economies	• 18 detailed household diaries, including gender differences (Figure 3)
		• 20 village and district leader interviews
		• Village stakeholder workshops
	Advanced degree training for African national	• Rwandan PhD, Ecological Economics (University of Vermont)
***Policy***	Develop new health and environmental policy interventions to mitigate the impacts of zoonotic diseases	• Strong partnerships with local governments, health and environment ministries, and policy and education NGOs
		• USAID policy briefs
		• Integrative modeling
	Raise awareness about the links among health, livelihoods, and natural resources	• Active participation in stakeholder meetings, international conferences, and ministry presentations
		• HALI Project blog
		• Public outreach through movie nights, radio programs, and zoonotic disease calendar

The HALI project has identified bovine tuberculosis and brucellosis in livestock and wildlife in the Ruaha ecosystem and is using this information to identify geographic areas with varying water availability where risk of transmission among wildlife, livestock, and people may be high. In addition, *Salmonella*, *Escherichia coli*, *Cryptosporidium*, and *Giardia* spp. that can cause disease in humans and animals have been isolated from multiple water sources used by people and frequented by livestock and wildlife. These data are now being used to examine spatial and temporal associations between landscape factors and disease and to identify risk factors and health impacts that may be mitigated through policy changes and outreach. Preliminary findings also indicate that more than two-thirds of participating pastoral households do not believe that illness in their families can be contracted from livestock, and nearly half believe the same of wildlife. Furthermore, when the HALI project began working in this region, 75% of households did not consider sharing water sources with livestock or wildlife a health risk, illustrating the need for effective community education.

## Lessons Learned for Planning One Health Projects and Interventions

The HALI platform has reinforced the importance of the One Health concept and provided lessons for the development of a new approach to global health.

**Figure 3:**
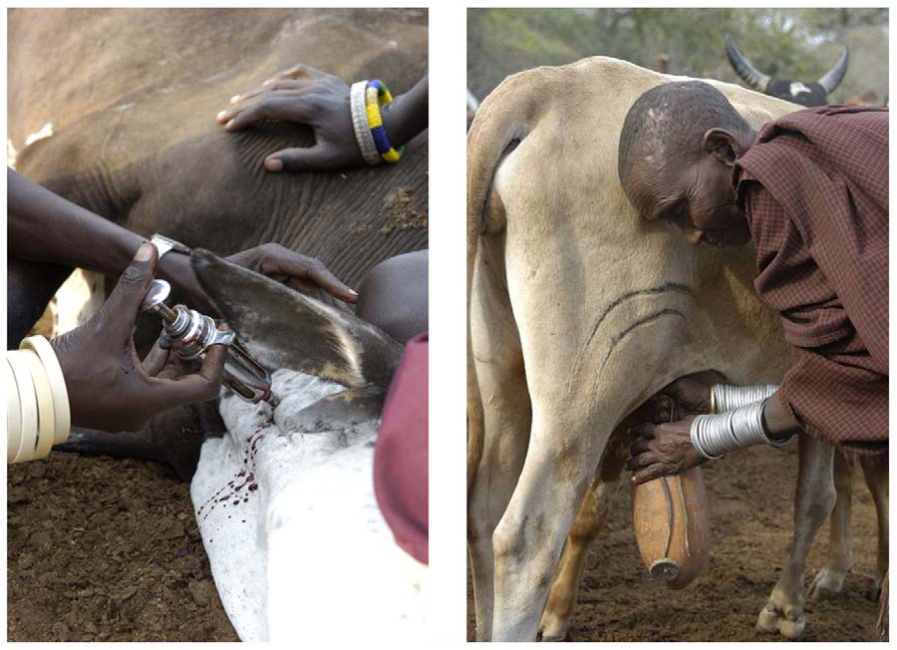
Men's and women's disease risks from livestock likely differ: men have occasional, but intense contact with sick animals (left), while women have regular, close contact with animals, particularly poultry and lactating cows and goats (right). (Photo credit: M. Kock-Wildlife Conservation Society).

First, it is crucial to recognize that zoonotic pathogens are present and emerging in rural communities and that their emergence is spatially and temporally variable within these communities. Most people living in high risk areas are not aware of the danger or what can be done to reduce it. In addition, transmission can be exacerbated by common animal husbandry and food and water handling practices (Figure 3) [23]. Therefore, data collection strategies should include the evaluation of spatial, temporal, and demographic patterns of pathogen prevalence and disease in human, domestic animal, and wildlife populations in likely hotspots for disease emergence. The underlying water- and land-use determinants of disease and the social, economic, and cultural barriers to control and prevention must be explored [24],[25]. While local stakeholders and international institutions actively involved in animal health, conservation, and livelihood assessment and improvement were quick to engage in HALI, physicians and public health experts (local and international) have been slower, likely due to competing demands on time and resources already dedicated to addressing malaria and tuberculosis of human origin [5]. Concerns over the financial escalation of projects directly measuring pathogens in humans was also an obstacle to engaging medical professionals for these neglected diseases.

Second, the role of water in disease transmission and zoonosis emergence should be further explored. Water scarcity increases work stress, especially in women and children, and brings animals and people together more frequently, increasing the likelihood of water contamination and transmission of infectious diseases. Likewise, the manner in which water is used for agricultural and animal production affects worker health, food safety, and the health of those who drink and bathe in it. Improving water safety and security, including sanitation, in ecologically appropriate ways that reduce disease risk will require a transdisciplinary approach in which economists, ecologists, epidemiologists, and engineers play important roles with public and animal health practitioners.

Finally, the determinants and consequences of zoonotic diseases, as well as the interventions to mitigate their deleterious effects, are all cross-sectoral. Effective surveillance, assessments, and interventions are possible only by bridging the organizational gaps among institutions studying and managing wildlife, livestock, water, and public health. It is clear that education in global health, especially emerging zoonotic diseases, is urgently needed at all levels from research institutions to pastoralist communities. Collecting detailed data regarding land use and agricultural practices, food consumption and water use habits, illness in animals and people, and access to health care will help appropriately tailor education efforts for priority diseases and pandemic prevention. The donor community should be encouraged to transcend disciplinary conventions and invest in holistic health projects that have the best chance of affecting change.

## Abbreviations

BTB: bovine tuberculosis
EPTB: extrapulmonary tuberculosis
HALI: Health for Animals and Livelihood Improvement
NGO: nongovernmental organization

## References

[1] World Health Organization (2009). Data and statistics: Causes of death.

[2] World Health Organization (2006). The control of neglected zoonotic diseases: A route to poverty alleviation.. http://whqlibdoc.who.int/publications/2006/9789241594301_eng.pdf.

[3] Taylor LH, Latham SM, Woolhouse MEJ (2001). Risk factors for human disease emergence.. Phil Trans Royal Society B.

[4] Jones KE, Patel NG, Levy MA, Storeygard A, Balk D (2008). Global trends in emerging infectious diseases.. Nature.

[5] Moran M, Guzman J, Ropars A-L, McDonald A, Jameson N (2009). Neglected disease research and development: How much are we really spending?. PLoS Med.

[6] Clifford D, Kazwala R, Coppolillo P, Mazet J (2008). Evaluating and managing zoonotic disease risk in rural Tanzania.. http://glcrsp.ucdavis.edu/publications/HALI/08-01-HALI.pdf.

[7] Coppolillo P, Dickman A, Redford KH, Fearn E (2007). Livelihoods and protected areas in the Ruaha Landscape: A preliminary review.. Protected areas and human livelihoods.

[8] Ogelthorpe J, Gelman N (2007). HIV/AIDS and the environment: Impacts of AIDS and ways to reduce them. A fact sheet for the conservation community.

[9] Newark WD (2008). Isolation of African protected areas.. Front Ecol Environ.

[10] Wittemyer G, Elsen P, Bean WT, Coleman A, Burton O (2008). Accelerated human population growth at protected areas edges.. Science.

[11] United Nations (2008). Contributing to One World, One Health: A strategic framework for reducing risk of infectious diseases at the animal-human-ecosystem interface.. http://un-influenza.org/files/OWOH_14Oct08.pdf.

[12] Osofsky SA, Kock RA, Kock MD, Kalema-Zikusoka G, Grahn R, McNeely JA (2005). Building support for protected areas using a “One Health” perspective.. Friends for life: New partners in support of protected areas.

[13] Ray JC, Hunter L, Zigouris J (2005). Setting conservation and research priorities for larger African carnivores.

[14] Tanzania Ministry of Agriculture and Food Security (2002). Agriculture Sector Development Strategy.

[15] National Bureau of Statistics (2002). Household Budget Survey 2000/01.

[16] Wolfe N, Daszak P, Kilpatrick AM, Burke DS (2005). : Bushmeat hunting: Deforestation, and prediction of zoonotic disease emergence.. Emerg Inf Dis.

[17] Kilonzo BS, Komba EK (1993). The current epidemiology and control of trypanosomiasis and other zoonoses in Tanzania.. Central African J Med.

[18] John K, Kazwala RR, Mfinanga GS (2008). Knowledge of causes, clinical features and diagnosis of common zoonoses among medical practitioners in Tanzania.. BMC Infect Dis.

[19] Cosivi O, Grange JM, Daborn CJ, Raviglione MC, Fujikura T (1998). Zoonotic tuberculosis due to *Mycobacterium bovis* in developing countries.. Emerg Inf Dis.

[20] National Tuberculosis and Leprosy Programme Tanzania (2006). Annual Report for 2006.

[21] Range N, Ipuge YA, O'Brien RJ, Egwaga SM, Mfinanga TM (2001). Trend in HIV prevalence among tuberculosis patients in Tanzania, 1991–1998.. Int J Tuberculosis Lung Dis.

[22] Kazwala RR, Daborn CJ, Kusiluka LJM, Jiwa SFH, Sharp JM, Kambarage DM (1998). Isolation of *Mycobacterium* species from raw milk of pastoral cattle of the southern highlands of Tanzania.. Trop Anim Health Prod.

[23] Mfinanga SG, Morkve O, Kazwala RR, Cleaveland S, Sharp JM (2003). Tribal differences in perception of tuberculosis: A possible role in tuberculosis control in Arusha, Tanzania.. Int J Tuberculosis Lung Dis.

[24] Patz JA, Daszak P, Tabor GM, Aguirre AA, Pearl M (2004). Unhealthy landscapes: Policy recommendations on land use change and infectious disease emergence.. Envir Health Persp.

[25] Kock MD, Osofsky SA (2005). The health paradigm and protected areas: linkages between people and their livelihoods, ecosystems and natural communities, and health and disease.. Conservation and development interventions at the wildlife/livestock interface: Implications for wildlife, livestock and human health.

